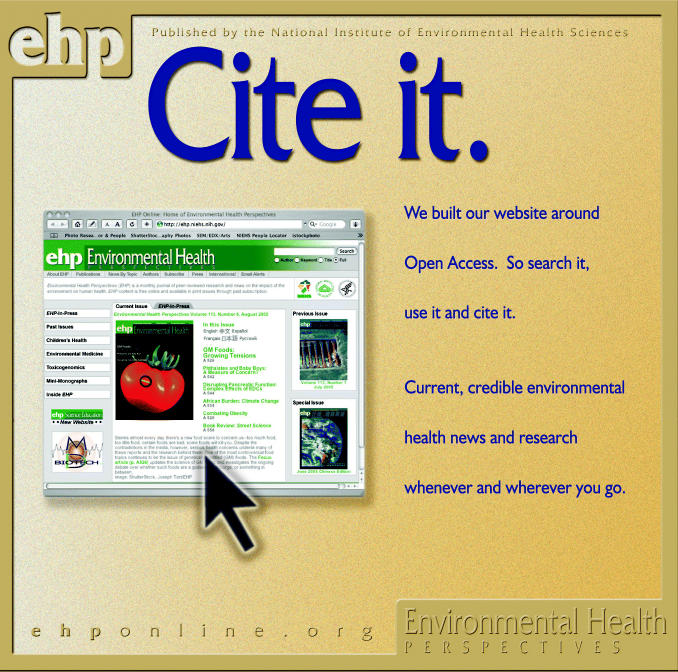# Harvesting the Potential of BIOMASS

**DOI:** 10.1289/ehp.113-a750

**Published:** 2005-11

**Authors:** David J. Tenenbaum

As fossil fuel prices and consumption both continue rising, the search is on for alternative fuels. Fuel for vehicles is taking center stage, now that 67% of U.S. petroleum consumption goes toward fueling vehicles, according to the U.S. Department of Energy (DOE). Could biomass energy derived from plant matter supply a significant percentage of future transportation fuel? The answer is yes, according to *Biomass as Feedstock for a Bioenergy and Bioproducts Industry: the Technical Feasibility of a Billion-Ton Annual Supply*, a report funded by the DOE and the U.S. Department of Agriculture (USDA) and issued by Oak Ridge National Laboratory in April 2005.

## Biomass Today

The report defines biomass as all plant-derived molecules, including grain, starch, sugar, oil, and waste products, as well as the plant structural components cellulose, hemicellulose, and lignin. Fossil fuels and animal matter are excluded. While the diversity of resources is a strength of biomass, it also raises a problem: different facilities are needed to convert the array of molecules from biomass into the hydrocarbons needed for transportation fuel. Furthermore, large changes in infrastructure would be needed to harvest the various potential sources.

The report gives an overview of the biomass situation today in the United States. About 190 million dry tons of biomass feedstock are consumed annually. Biomass accounts for about 3% of total U.S. energy supplies, and has recently surpassed hydropower as the largest renewable energy source. A great deal of biomass is waste material that is both produced and consumed by industry. For example, forest-products firms (including paper companies) use 96 million dry tons of biomass, largely to power their factories. Currently, 3.4 billion gallons of ethanol are blended into gasoline each year; that amount could soar to 80 billion gallons by 2030.

The report states that by 2030, American acreage could produce enough biomass to displace at least 30% of the country’s current consumption of petroleum fuels with some changes in land use and agricultural and forestry practices, and up to 50% with advanced conversion technologies. This calculates to up to 1.366 billion tons of biomass produced annually.

Lynn Wright, who formerly worked in Oak Ridge National Laboratory’s biomass program and now consults to it, says the report was a response to a common question from the energy industry. “We were hearing from people with connections to oil companies that they hardly considered it worth their while to think about biomass unless we could show that it could supply as much as a billion dry tons per year,” she says.

The *Billion-Ton* report does not attempt to assess the economic viability of large-scale biomass energy, and Wright explains that it’s difficult at best to predict the relative price of fossil fuels and the various sources of biomass in 25 years. Today, she says, when prices are measured by energy content (British thermal units), energy from switchgrass or corn stover, the residue that remains in the field after a grain crop is harvested, is cheaper than energy from oil but more expensive than energy from coal.

## Boosting Production

According to the report, wood could grow to supply 368 million dry tons of biomass by 2030. The supply could expand with enhanced collection of urban tree trimmings and construction waste, and greater efforts to prevent forest fires by clearing deadwood from forests.

But transportation and processing costs may keep wood expensive, cautions report coauthor Bryce Stokes, program leader of vegetation management and protection research at the USDA Forest Service. He says, “We still have to overcome some economic and conversion efficiency barriers . . . to make wood competitive” in transportation fuels. Woody biomass will seem more competitive, he adds, if the benefits of improving forest heath, reducing fire risk, and recycling carbon from the atmosphere are held in view.

Farms could potentially contribute a far larger quantity of biomass (998 million dry tons), and much of that may come from corn stover and perennial crops managed with no-till production techniques and collected with advanced harvesting equipment. However, Wally Wilhelm, a plant physiologist with the USDA Agricultural Research Service, says it is unlikely that all land will ever be switched to no tillage. “Use of no-tillage methods and producing crops without tillage is far more complex than simply not passing over the field with a plow or disk,” he explains. “It takes time and skill, and trial and error, to become proficient at no-till farming. Not all farmers are willing, nor have the flexibility—the money in the bank—to pursue the knowledge and skill.”

Corn grain is currently the source of most of the ethanol used as motor fuel in the United States today. Corn production has been growing by 1.7 bushels per acre per year for 30 to 40 years, says Achim Dobermann, a professor of soil science and nutrient management at the University of Nebraska. Dobermann says irrigated cornfields in Nebraska could produce 250 to 350 bushels per acre.

These yields require intensive inputs, especially in the form of nitrogen fertilizer, which is usually derived from natural gas. Farmers already manage nitrogen closely, due to its price and potential for polluting groundwater, but ever-higher yields will force them to work even harder to carefully manage nitrogen. “It requires a more fine-tuned type of management,” says Dobermann. “You can’t just go in and apply anhydrous ammonia [a common nitrogen fertilizer] in the fall and take off for vacation.” Instead, he suggests multiple nitrogen applications, timed and placed when and where the crop needs it.

## More Mass, Sustainably

If biomass harvesting is to be sustainable, it must not diminish soil’s fertility (its ability to supply nutrients for plant growth) or other properties influencing productivity. A market for stover creates an incentive for farmers to remove more after the harvest. But crop residue left in the fields reduces soil erosion; it also improves soil fertility and structure through the addition of organic carbon, which fuels microbial activity that drives the cycling of nutrients and structures in productive lands.

Estimates of how much stover must remain on the fields if erosion is to be controlled rely on the concept of a tolerable amount of soil loss, as defined for particular soils by the USDA Natural Resource Conservation Service. But this amounts to “an educated guess,” says Wilhelm. “The assumption is that if we keep losses below the tolerable level, we should not notice a significant impact on productivity.” Erosion is affected by farming practices, soil types, and weather, and Wilhelm says it’s “a very good question” whether it’s possible to predict what level of stover removal will hold soil organic carbon loss below tolerable levels.

Because crop residue is converted into organic matter that maintains soil structure, Wilhelm says levels of organic matter may be a good metric of soil health and the amount of stover that must be retained on or in the soil to sustain productivity. Wilhelm, who is leading a project to develop guidelines for sustainable removal of corn stover, says extensive biomass extraction raises the danger that soil organic carbon will be “mined” rather than be treated as the irreplaceable resource that it is. He adds that stakeholders must work together to develop systems that enhance the use of renewable sources of energy and produce renewable energy in a sustainable manner.

## A Question of Impact

One of the key arguments over biomass energy concerns the net energy contribution of biomass—how much energy is gained from the crop. For example, David Pimentel, a professor of ecology and agriculture at Cornell University, and Tad Patzek, a professor of civil and environmental engineering at the University of California, Berkeley, published calculations in the March 2005 issue of *Natural Resources Research* showing that ethanol derived from corn contains only 71% of the energy used to grow, harvest, and convert the grain into ethanol. At the other end of the spectrum, calculations by federal researchers Hosein Shapouri, James A. Duffield, and Michael Wang in the July 2002 *Agricultural Economic Report Number 814* showed a net energy gain of up to 130–140%.

Pimentel and Patzek based their calculations on average U.S. corn production output for 2003—140 bushels per acre. As to the assertions put forth in the *Billion-Ton* report, Pimentel contends that providing enough biomass to cover 30% of current U.S. gasoline and diesel use would require a land area greater than that of the United States. He believes the actual U.S. biomass capacity is about half the 1.366 billion tons cited in the report.

But calculating net energy efficiency is difficult, says Robert Anex, an associate professor of agricultural and biosystems engineering at Iowa State University who studies life-cycle assessments of biomass resources. “One must account for all of the resources that are used, all of the product created, and also those resources that are saved via substitution of the biomass product for some other probably petroleum-based product,” he says. “This involves many assumptions about how crops are grown, harvested, and converted, but also what resource use is avoided.” This, he says, is why these sorts of measures are often contentious.

Biomass advocates such as Thomas Foust, biomass program technology manager at the DOE National Renewable Energy Laboratory, say more biomass should become available if agricultural productivity continues its steady rise and improvements in conversion technologies are made—for example, ethanol production per bushel of corn has grown by about 25% in the past 25 years. Furthermore, Foust says that net energy balances for ethanol are not that useful, and the real metric should be imported oil displacement, which can be as high as 6 to 1 for ethanol.

The environmental health impact of gathering 1 billion tons of biomass through whatever means—a plan that could affect hundreds of millions of acres—must also be investigated thoroughly. For example, the impact of harvesting biomass from millions of acres of farmland now set aside under the USDA Conservation Reserve Program remains to be studied. One higher-production scenario in the *Billion-Ton* report assumes that 60 million acres would be shifted from a combination of Conservation Reserve Program land, pasture land, and commodity crop production in order to produce woody and grass crops as a source of biomass. The land used to produce wood and grass crops would provide bird and mammal habitat similar to the Conservation Reserve Program but would be harvested more frequently.

Donald Waller, a professor of botany and environmental studies at the University of Wisconsin–Madison, raises other questions about the impact of boosted biomass energy production on forest health. While noting that the report does not call for building roads in roadless forests or removing biomass from wilderness areas, he warns of broad ecological consequences from removing massive amounts of tree biomass and thus essential nutrients. “In most forests, the old growth is dominated by decomposers in terms of species number and complexity,” he says. “Deadwood is there in far greater quantity than live wood.”

Waller emphasizes the importance of leaving behind “biological legacies”—standing dead trees, live trees, and tree material on the ground. “You can’t take it all away without seriously diminishing the ecosystem functions and the plant and animals that live there,” he says.

## Achieving Critical Mass

In the face of tight fossil fuel supplies, the federal government is moving ahead with plans to expand biomass output. The National Renewable Energy Laboratory, for example, has a considerable effort working to improve biomass conversion into liquid fuel. Wright suggests putting more effort into pilot projects that use large amounts of biomass. “I think it would help a great deal to get some demonstrations in place on the part of farmers and power producers,” she says.

Government subsidies similar to the tax credits already offered to build wind power towers and install solar energy panels may be another way to enhance the appeal of biomass. In the 17 October 2005 issue of *Newsweek*, Frances Beinecke, executive director and incoming president of the Natural Resources Defense Council, says, “We think subsidies or assistance from the federal government should go to the new technologies that need to come to the market. . . . Biofuels are definitely part of the renewables portfolio. There’s growing interest in the agricultural sector, because that way we could have home-grown fuels.”

The energy business may be at a turning point. After years of concern about funding levels for alternative energy research, the prices of oil and natural gas have changed the equation, says Wright: “If prices stay high, I don’t think the government will have to do very much [to jumpstart the biomass bandwagon].”

## Figures and Tables

**Figure:**
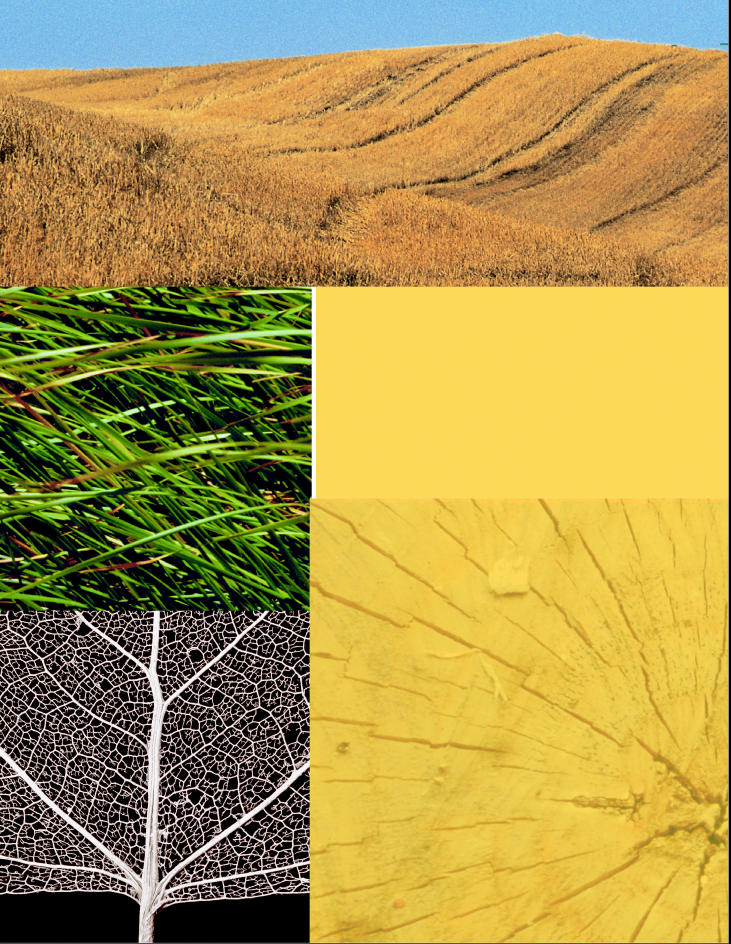


**Figure:**